# Passively Addressable Ultra-Low Volume Sweat Chloride Sensor

**DOI:** 10.3390/s19204590

**Published:** 2019-10-22

**Authors:** Antra Ganguly, Shalini Prasad

**Affiliations:** Department of Bioengineering, University of Texas at Dallas, 800 West Campbell Road, Richardson, TX 75080, USA; Antra.Ganguly@utdallas.edu

**Keywords:** wearables, sweat chloride sensor, continuous monitoring, non-faradaic electrochemical impedance spectroscopy, chronoamperometry, chloride ionophore

## Abstract

This work demonstrates a novel electrochemical biosensor for the detection of chloride ion levels in ultra-low volumes (1–3 microliters) of passively expressed human sweat. We present here a hydration monitor that the pediatric, geriatric, and other immune-compromised or physically inactive/sedentary population cohort can utilize, for whom the current methods of chloride quantification of active stimulation of sweat glands through iontophoresis or treadmill runs are unsuitable. In this work, non-faradaic electroanalysis using gold microelectrodes deposited on a flexible nanoporous substrate, for high nanoscale surface area to volume enhancement, was leveraged to operate in ultra-low sweat volumes of <3 µL eluted at natural rates. The specific chloride ionophore-based affinity of chloride ions resulted in the modulation of charge transfer within the electrical double layer at the electrode–sweat buffer interface, which was transduced using electrochemical impedance spectroscopy (EIS) and chronoamperometry (CA). Linear calibration dose responses with R-squared values of 0.9746 and 0.9403 for EIS and CA respectively were obtained for a dynamic range of 10–100 mM. The surface charge and the binding chemistry of the capture probe were studied using zeta potential studies and UV-Vis. The dynamic sweat chloride-tracking capability of the sensor was evaluated for a duration of 180 min. Studies were conducted to probe the efficacy of the developed sensor for passive ultra-low sweat chloride assessment on human subjects (n = 3).

## 1. Introduction

An individual’s physiological well-being can be mapped by examining the biomolecular makeup of their body fluids such as blood, urine, tears, saliva, and sweat [1,2,3]. The detection and quantification of these biomarkers, which may be proteins, hormones, metabolites, or ions in these body fluids, afford us diagnostic opportunities for their respective diseases. Among all these biofluids, sweat is largely considered as a suitable choice mainly because of its easy accessibility, simple sample preparation, and low sample adulteration susceptibility [3,4,5,6]. Furthermore, the non-invasive nature of sweat sampling makes it an ideal candidate biofluid for biomarker detection for diseases that require frequent monitoring and analysis by eliminating the risk of surgical infection [3,7].

Dissolved in all biofluids are charged species, which lend them their mineral content and are involved in vital physiological processes ranging from the regulation of neuromuscular function to maintaining the acid–base balance in the body [8]. These electrically charged minerals are called electrolytes. A healthy composition of electrolytes in the body results in water homeostasis. One of the most common cases of electrolytic imbalances is observed as dehydration, an acute condition which, in the simplest sense, is the deficiency of water from the body. One of the electrolytic biomarkers in sweat that can be tracked to quantify hydration levels is chloride [9,10]. Being the most abundant anion in the extracellular fluid matrix makes sweat chloride ions more suitable than cations such as sodium and potassium for tracking hydration levels with high specificity without ionic crosstalk. On the other end of the spectrum, the tracking of chloride levels in sweat are implicated in the diagnosis and prognostic tracking of chronic diseases such as cystic fibrosis [11,12,13].

There are a number of sensors for the detection of chloride in sweat that are available commercially or in the research space, which require sweat volumes in tens of microliters for reliable sensing [14,15,16,17,18,19]. This necessitates active addressing of sweat, i.e., the sweat has to be actively stimulated by exercise (treadmills, exercise bikes, etc.) in order to obtain a measure of the chloride content, which is unsuitable for use in physically inactive individuals or generally sedentary populations and individuals on chronic medication [20,21]. Alternatively, in immobile and immunocompromised individuals and in pediatric and geriatric populations, sweat is typically stimulated through electrical iontophoresis mechanisms or with the use of chemicals, both of which have shown deleterious effects on the epidermis [22,23]. The 2019 Physical Activity Council’s Overview Report on U.S. Participation reported that since 2013, more than a quarter of the US population greater than age 6 is physically inactive. In 2018, 82.1 million Americans were involved in activities with low to no physical exertion (inactivity rate 27.3%). Physical inactivity is also directly correlated with income. Since 2015, a lack of awareness or access to healthy unprocessed food option and lifestyle choices (gym membership, personal trainers, race entrance fees, etc.) has resulted in physical inactivity in 1.7 million people in the US.

The proposed sensor aims to cater as a hydration monitoring device to the above-mentioned population cohort by passively detecting chloride levels in 1–3 µL volumes of sweat. The natural eccrine sweat rate range is 1–5 nL/min/gland. Concomitant to this natural sweat rate and the typical eccrine sweat gland density of 100 glands/cm^2^, the active sensing area was optimized. To our knowledge, this work is the first demonstration of a passive sweat-based diagnostic device for tracking chloride ions in sweat in a non-faradaic manner. This device can sample sweat produced by the epidermis in a passive manner and in “real time”, and can report the chloride ion concentration. The sensor’s analytical metrics were first evaluated on the bench, and testing on human subjects was performed.

**Figure 1:**
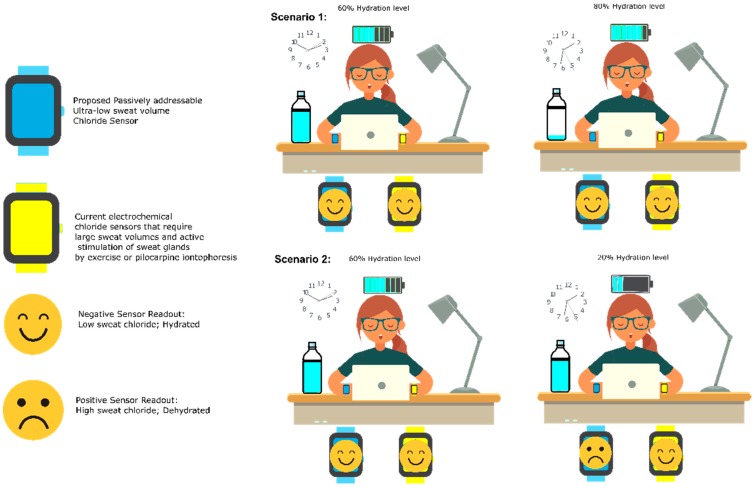
The proposed sensor requires sweat volumes related to natural sweating rates under normal ambient conditions; it has superior sensitivity compared to the currently available sweat chloride-sensing schemes.

Sweat chloride measurement techniques that are suitable for incorporation in wearables are broadly classified into three groups viz., titration-based devices, conductometric devices, and potentiometric devices [24,25]. Titration-based devices are slow, require post-processing, long wait times, and large analyte volumes. Conductometry measures the conductivity of the bulk ions in the sweat and are non-specific to chloride ions, while the potentiometric devices, though specific, suffer from poor sensitivity due to issues in equilibration between the test and reference solutions [25]. Figure 1 illustrates that since the proposed sensor requires sweat volumes related to natural sweating rates under normal ambient conditions, it has superior sensitivity compared to the currently available sweat chloride-sensing schemes. This was achieved by leveraging the highly sensitive capacitive transduction and enhanced nanoscale surface to volume enhancement. Nanoporous polyamide was used as the substrate for electrode deposition to achieve a high surface area to volume ratio in the active sensing region. In this work, we use non-faradaic electrochemical impedance spectroscopy (EIS), a simple yet powerful capacitive AC measurement technique, and non-faradaic chronoamperometry (DC current-based technique), which operate with high sensitivity and greater resolution for the slightest mM changes in chloride concentration. Among these two techniques, we found that the dielectric measurement obtained through EIS affords us a finer calibration and a better resolution for characterizing ions in sweat. Furthermore, concentration-dependent RC time constant measurements reveal that EIS has a comparatively greater dynamic range as compared to chronoamperometry, which means that the former allows for longer time to capture the dynamics of binding for a given perturbation. Both the techniques have a fast response times and dynamically express the sweat chloride content in less than 30 min for ultra-low volumes of passive sweat under standard ambient room temperature and relative humidity conditions. The proposed sensor leverages the high sensitivity of capacitive transduction for detecting chloride ion levels in sweat. The proposed method is advantageous, as it allows for non-faradaic sensing using a simple two-electrode electrochemical setup, which allows for a simplistic yet reliable dielectric modulation-based readout.

## 2. Materials and Methods

Polyamide substrates (pore size, 200 nm; thickness, 60 µm) were obtained from GE Healthcare Life Sciences (Piscataway, NJ, USA). The crosslinking molecule dithiobis succinimidyl propionate (DSP) and its solvent, dimethyl sulfoxide (DMSO), were purchased from Thermo Fisher Scientific Inc. (Waltham, MA, USA). The chloride ionophore, 4,5-Bis-[N′-(butyl) thioureido]-2,7-di-tert-butyl-9,9-dimethylxanthene (synonym: Chloride Ionophore IV Selectophore™), was purchased from Sigma-Aldrich (St. Louis, MO, USA). Ionophore solution was prepared by dissolving it in DMSO and vortexing it for 30 min at 1000 rpm. Perspired human sweat of pH 5.98 from a single donor was obtained from Lee Biosolutions Inc. (Maryland Heights, MO, USA) and was stored at −20 °C, without the addition of any preservatives. Potassium chloride, which was used to spike the human sweat samples with the desired chloride levels for sensor calibration, was ordered from Thermo Fisher Scientific Inc. Synthetic sweat (pH 2, 4, 6, and 8) was used as a buffer for zeta potential studies, which was prepared as per the recipe described by Mathew et al., minus the NaCl and KCl dissolution [26]. Buffer pH was varied using lactic acid (85%) and urea, which were obtained from Sigma-Aldrich (St. Louis, MO, USA) and Thermo Fisher Scientific Inc. (Waltham, MA, USA), respectively.

### 2.1. Sensor Fabrication

**Figure 2:**
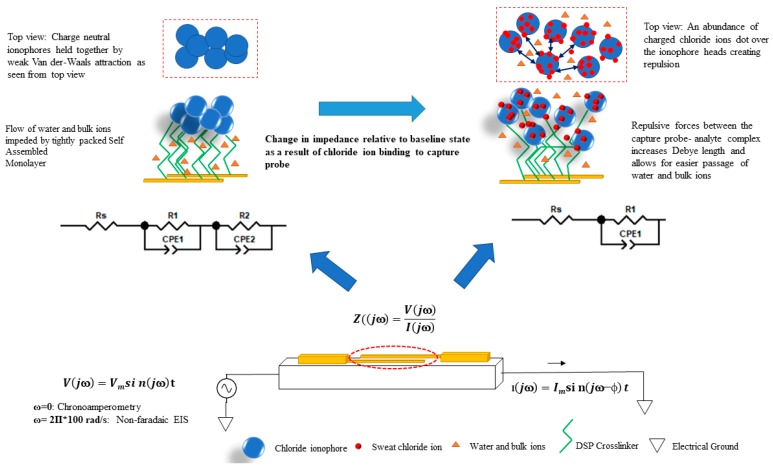
The proposed chloride sensor device can be used to measure chloride levels in ultra-low volumes of passively addressed sweat. Gold working and reference/counter microelectrodes have been deposited on the nanoporous polyamide substrate. The sensor stack consists of a chloride ionophore, a neutral organic bioinert molecule, to which sweat chloride ions preferentially bind through strong hydrogen bonds.

The proposed sensing scheme involves a standard two-electrode electrochemical system, as shown in Figure 2. Both the working and reference/counter electrodes are made up of gold. Cryo e-beam evaporation, using a shadow mask, was used to deposit the gold electrodes, of 1.5 kA° thickness, onto the flexible nanoporous polyamide substrate at an average deposition rate of ~1 A°/s. The final thickness of the deposited electrodes was verified using a Veeco Dektak 8 profilometer [27].

### 2.2. UV-Vis for Validation of Binding Chemistry

The binding chemistry was further probed by UV-Vis analysis of the chloride ionophore–DSP complex using DMSO as a blank buffer/control. The scan was performed for the wavelength range of 200–800 nm using a Cary 7000 (Instrument Version 2.24).

### 2.3. Zeta Potential Measurements for Validation of Electrical Neutrality of Capture Probe

In order to assess the surface charge variation of the chloride ionophore in the entire physiological range of sweat pH (2, 4, 6, and 8), the zeta potential (ζ) of the chloride ionophore was measured. Zeta potential measurements of 30 mM chloride ionophore in synthetic sweat of varying pH values of 2, 4, 6, and 8 were performed using the Malvern Zetasizer NanoZS (Malvern Instruments Ltd., Malvern, Worcestershire, UK). Diffusion barrier technique was used to determine the electrophoretic mobility of the ionophore in the buffer, which was translated to its zeta potential for a given buffer pH using Smoluchowski approximation [28,29].

### 2.4. Sensor Calibration in Perspired Human Sweat for Chloride Sensing

**Figure 3:**
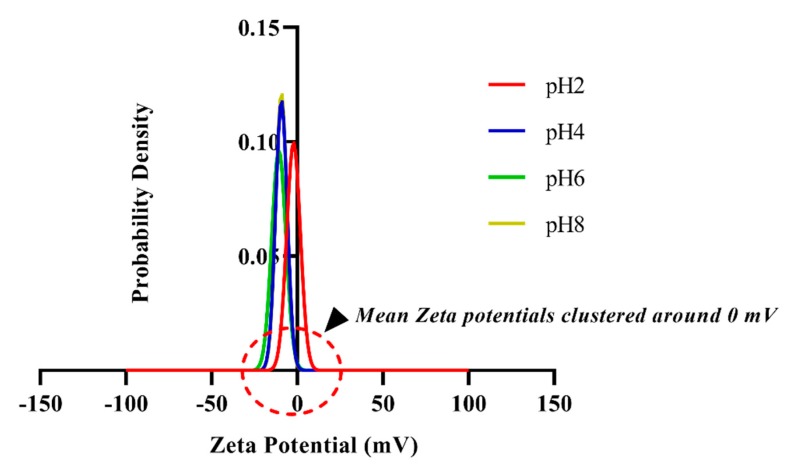
Probability density functions of zeta potential values for the entire physiologically relevant pH range (pH 2, 4, 6 and 8) measured in a human sweat analogue by the diffusion barrier method. Neutral or near-neutral behavior is evident from the clustering of the pdfs being 0 mV.

The label-free sensing assay has been depicted in Figure 2. The proposed biosensing scheme relies on the affinity capture chloride ions in ultra-low volumes (1–3 μL) of human sweat. The chloride ionophore, 4,5-Bis-[*N*′-(butyl)thioureido]-2,7-di-tert-butyl-9,9-dimethylxanthene (commercially known as Chloride Ionophore IV) serves as the capture probe for the proposed affinity biosensor to which the chloride ions in sweat preferentially bond by way of strong hydrogen bonding [30]. This capture probe is crosslinked to the gold electrode surface by a self-assembled monolayer of DSP. The ordered and desirably spaced self-assembly of DSP molecules on the electrodes is achieved by taking advantage of the strong thiol bonds formed between DSP and gold. In a chloride ionophore IV molecule, there exist four possible H-bond donor sites with which the ionophore can non-covalently interact. The polyamide substrate is appreciably hydrophilic and allows for the quick and uniform wicking of ultra-low volumes of both human sweat (aqueous) and DMSO (polar aprotic solvent) onto the surface without forming a heterogeneous mixture. The selected ionophore possesses a highly preorganized bis-thiourea receptors-based xanthene spacer which captures sweat chloride ions by multitopic hydrogen bonding with very high specificity. The stochiometric ratio of chloride ionophore to DSP (404.42 g/mol) has been kept to 3:1, which allows for sufficient binding sites for the target chloride ions. The chloride ions (<35.453 g/mol) are negligible in size when compared to the bulky ionophores (~582.91 g/mol). This small size allows them to diffuse swiftly to the electrode by Fick’s Law and be reliably captured as they bump into the sufficiently large ionophore molecules. Furthermore, since the chosen ionophore is electrically neutral, as validated by the results of our zeta potential studies (Figure 3), the possibility of steric hinderance and charge screening due to the high density of ionophore sites is not an issue here. It is proposed that chloride ionophore is strongly bound to the of the DSP molecule by way of a binding chemistry that closely imitates the NHS ester bonds with secondary amines.

First, 10 mM of DSP was diluted in DMSO and wicked on the polyamide substrate of the sensor for an incubation time of 2 h. This was followed by dispensing of 3 µL of 30 mM chloride ionophore (dissolved in DMSO) on the sensor. The incubation time for chloride ionophore was optimized to 2 h and was found to be sufficiently large for the presumed secondary amine NHS ester binding to take place before the effect starts to saturate. Then, 3 µL of human sweat (pH ~6) was dispensed on the sensor surface. This was the baseline or the zero dose. In healthy individuals, the chloride levels for adults and infants are <40 mM in adults and <30 mM, respectively [7,31,32,33]. Since the human sweat was procured from a single healthy donor, it is safe to assume that the sweat chloride levels are well below the 30 mM bookend for CF or chloride fluctuation anomaly screening. To calibrate the sensor, a dynamic range of 10–100 mM was aimed for. Potassium chloride was dissolved in the blank/zero dose human sweat to obtain 10, 20, 40, 50, 80, and 100 mM chloride concentrations for sensor calibration. The chloride-spiked sweat was incubated on the sensor for 30 min. This was followed by a chronoamperometry (CA) measurement for a step voltage of 0.5 V for 5 s and a potentiostatic, non-faradaic EIS run by applying a 10-mV signal for a frequency sweep from 1 Hz to 1 MHz (Gamry Instruments, Warminster, PA, USA). To our knowledge, the selected chloride ionophore molecule has not been used for EIS measurements; thus, in order to account for all possible behavior, a huge spectrum was analyzed. Small signal non-faradaic EIS is an AC-based technique, while non-faradaic chronoamperometry (response similar to a discharging capacitor) is a DC-based method, both of which can be easily translated from the confines of benchtop electroanalysis to a more portable handheld POC or wearable device.

A sample size of n = 5 for EIS and n = 4 for CA was used to calibrate the sensor against the different physiologically relevant concentrations of sweat chloride. The sensor response was reported as the percentage change in the modulus of the impedance value measured at 100 Hz from the EIS data for a given concentration. Upon analyzing the entire frequency spectra from the LF range (1 Hz) to VHF range (1 MHz), it was found that the greatest signal-to-noise ratio was achieved at 100 Hz in the sense that the chloride ion capture probe activity was the most pronounced at this frequency point. Both the EIS and CA calibration dose–response (CDR) plots were obtained by fitting the respective data using polynomial curve fitting and the regression analysis coefficient (R^2^) evaluation. All the data are represented as mean ± standard deviation (SD).

### 2.5. Continuous Monitoring of Chloride Ions in Human Sweat

The dynamic response of the sensor was studied by the continuous dosing and monitoring of chloride levels in human sweat (n = 3). First, 10 mM (low), 50 mM (medium), and 100 mM (high) chloride doses in human sweat (pH 5.98) were dispensed on the sensor in volumes of 3 µL for 3 h. Each dose was wicked 3 times, with a time lapse of 20 min between successive doses, which was followed for the next higher doses i.e., 9 dispenses in 180 min (or 3 h). The sensor response was evaluated in terms of the percentage change in the imaginary part of the impedance value relative to the baseline value (blank human sweat solution at time = 0 s) at 100 Hz. Polynomial curve fitting and the regression analysis coefficient (R^2^) evaluation were performed and reported. All the data are represented as mean ± standard error of mean (SEM). All statistical analyses were done using statistical and graphing software Graph Pad Prism version 8.1.1 (Graph Pad Software Inc., La Jolla, CA, USA).

### 2.6. Sweat Chloride Sensing on Human Subjects

On-body tests were performed on 3 human subjects for three technical replicates. The sweat chloride measurement on human subjects was conducted in strict compliance following a protocol approved by the Institutional Review Board (IRB) at the University of Texas at Dallas, Richardson, TX, USA. The sweat chloride concentrations were probed by leaving the sensor functionalized with the assay on the subject’s right forearm for 30 min to allow the sweat chloride to interact with the capture probe. This was followed by mounting the sensor on the benchtop equipment and running CA and EIS measurements. The CA and EIS values measured after the chloride ionophore wicking step were considered as the baseline for these experiments. The normalized modulus of impedance value at 100 Hz and the chronoamperometric peak current value relative to the baseline were reported. 

## 3. Results

### 3.1. Binding Chemistry and Working of the Sensor Stack

**Figure 4:**
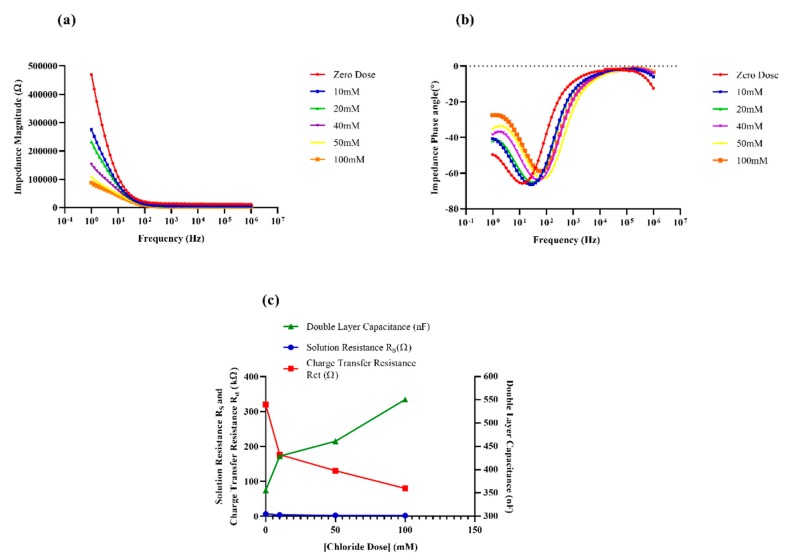
(**a**) Bode magnitude and (**b**) Bode phase plots obtained from non-faradaic electrochemical impedance spectroscopy (EIS) measurements for 1 Hz to 1 MHz range. Equivalent circuit parameters for the circuit corresponding to chloride dosing (Figure 2) were extracted for χ^2^ ~0.001 are shown in (**c**).

The proposed sweat chloride sensor is in the form of a two-electrode electrochemical cell. It comprises of gold working and reference (or counter) microelectrodes deposited on a flexible, hydrophilic substrate where the counter electrode also serves as the well-poised reference electrode that is maintained at relatively constant potential. Open circuit potential (OCP) measurements of the electrode system revealed that the sensor response stabilizes at 1500 s after the electrode is introduced to the aqueous environment [see Supplementary Material]. This is associated with the inherent potential gradient and is indicative of time after which a dynamic equilibrium is reached. The magnitude of the measured OCP was found to be in the lower millivolt range (35.8 mV), which is typical of a stable electrochemical sensor [34]. The stacking of DSP on the gold electrodes and that of the chloride ionophore on the DSP crosslinker resulted in composite electrodes [34]. The interfacial behavior of these composite electrodes and the ambient sweat buffer, in response to various chloride dose concentrations, was probed using non-faradaic EIS and non-faradaic chronoamperometry. Electrochemical impedance spectroscopy is a powerful AC-based electroanalytical sensing method in which a small sinusoidal signal (generally voltage) is applied by a potentiostat in an electrochemical cell containing the electrolytical solution (spiked with the analyte), and the resultant current at the working electrode is measured [34,35]. Based on the magnitude and phase lag of the resultant current, the potentiostat then calculates and reports the impedance value in terms of its real and imaginary components. This process is repeated over a range of frequencies to capture the behavior of the interfacial region over a large spectral range: 1 Hz to 1 MHz in this work [36,37,38]. Non-faradaic EIS is the impedance spectroscopic technique wherein there is a modulation in the attributes (capacitive changes) of the electrode–solution interface with no interfacial charge transfer. Non-faradaic EIS biosensors are often viewed as capacitive biosensors, and since no additional redox reagent is used in the sensing procedure, they have garnered considerable traction in POC and wearable development [35]. The results of EIS are as Bode magnitude and phase plots, where the magnitude and the phase of the interfacial impedance are plotted as a function of frequency. The interaction between the charged electrode and the sweat buffer creates an electrical double layer and the biomolecular crosslinker-capture probe stack creates the surficial capacitance. The chloride dosing response was analyzed using perspired human sweat samples (pH ~6) to calibrate the sensor for both EIS and CA. The variation in chloride dosing was achieved by the addition of potassium chloride into the sweat sample. Initial studies were based on synthetic sweat (SS pH 6, without KCl and NaCl) wherein the Nyquist and Bode plots for the blank solution (or zero dose) and that of the spiked solutions were compared [see the Supplementary Material]. The Nyquist plot for the SS zero dose consists of a small semicircle and a long diffusional tail, which are indicative of a small charge transfer resistance in the mid–high frequency range and the easy diffusion of sweat ions and metabolites (lactate, ammonia etc.) through the gaps between the head groups of the ionophores in the low-frequency range. It is speculated that if a nanoscale snapshot of the sensor stack were to be captured from the top, it would appear as a blanket of bulky ionophoric spheres balanced onto the gold electrodes via the DSP crosslinker. This blanket-like structure would be largely packed, leaving tiny pores/holes, because the bulky ionophore headgroups would rearrange themselves in such a way that affords them minimum steric hinderance and maximum Van der Waal’s attraction forces. When spiked sweat is transported onto the sensor, an abundance of chloride ions is introduced in the electrode–electrolyte system. These ions preferentially adsorb to the relatively large ionophore surfaces. An abundance of charged chloride ions dotting the ionophores produce significant repulsive forces to push them away, thereby dilating the pores on the “ionophore blanket” (Figure 2). This considerably reduces the transfer of bulk ions and water molecules, resulting in the decrease in the charge transfer resistance R_ct_, as evidenced by the reduction in the semicircle of the Nyquist plot with chloride dosing [see Supplementary Material]. On comparing the fitted equivalent electrical circuit (χ^2^ ~0.001) representative of the electrode–electrolyte interface after the ionophore incubation step and that of the first chloride dose (KCl in ultra-pure DI water), a switch in the circuit from two parallel RC circuits to a single parallel RC circuit was observed (Figure 2), indicating that the effects of the DSP–ionophore binding and the electrode bulk ion capacitance were overridden by the stronger influence of the chloride ions binding to the capture probe [see Supplementary information]. This effect is also observable in the shift observed from zero dose to the 10-mM dose in the Bode phase plot in Figure 4b.

For the spiked human sweat samples (pH 5.98), a similar dose-dependent decrease in R_ct_ was noted. When the negatively charged chloride ions bound to the capture probe, an increase in the accumulation of the charges in the double layer was manifested as the increase in the capacitance of the surficial capacitor (denoted by Cdl for double layer capacitance). As expected, an ingress of chloride ions in the bulk solution also cause a drop in the overall solution resistance Rs in a dose-dependent fashion. The Bode plots clearly represent the dose-dependent modulation of the double-layer reactance. This interfacial impedance modulation by EIS is modeled using complex nonlinear least squares (CNLS) fitting of an equivalent Randle’s circuit (χ^2^ ~0.001) using Zview software Version 3.5f (Scribner Associates Inc., Southern Pines, NC, USA) as shown in Figure 4c. The fitted circuit assumes the nature of a typical Randle’s cell with the Rs being in series with the parallel combination of R_ct_ and C_dl_. As expected, a dose-dependent decrease in solution resistance and charge transfer resistance R_ct_ and an increasing in the double-layer capacitance C_dl_ was observed with chloride dosing.

In addition to EIS, chronoamperometry was also performed, wherein a voltage pulse is applied at the working electrode to charge the non-faradaic capacitor and the relaxation of the double layer is gauged by the peak value of the exponentially decaying transient current. The peaks of the chronoamperometric currents were directly correlated to the chloride doses, which indicates that the charge accumulated in the double-layer capacitor (area under the chronoamperogram) was modulated by the binding of the chloride ions to the ionophore [see Supplementary Material].

**Figure 5:**
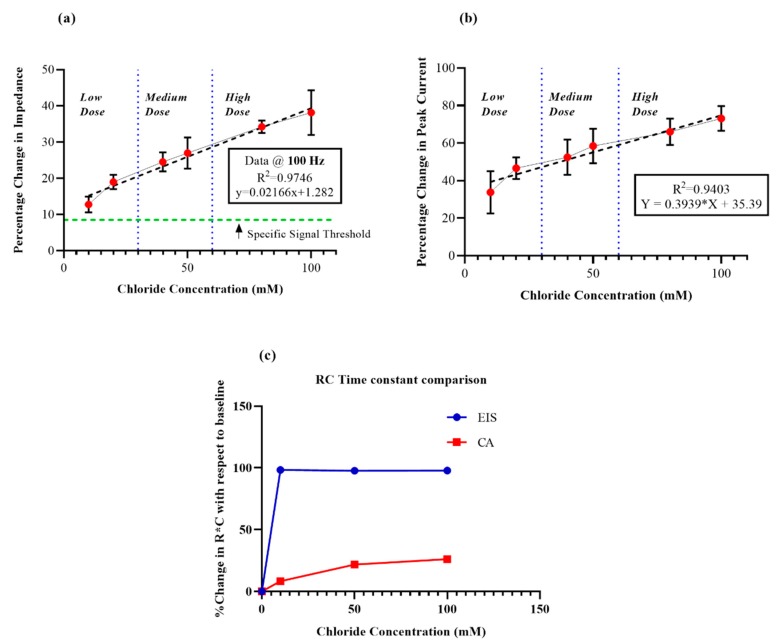
(**a**) Calibration dose response for non-faradaic EIS (100 HZ) for low, medium, and high chloride doses (n = 5), (**b**) Calibration dose response for non-faradaic chronoamperometry (CA) for low, medium, and high chloride doses (n = 4), (**c**) Comparison between the time constant (product of R_ct_ and C_dl_) of the EIS data and the average discharging time constant of the chronoamperograms.

Upon investigation and comparison between the time constant (product of Rct and Cdl) of the EIS data and the average discharging time constant of the chronoamperograms (Figure 5c), it was found that EIS showed a larger dynamic range for the time constant, meaning that the capacitive method (EIS) of probing affords a comparatively larger output range and a longer dwell time to capture the interfacial modulation behavior.

UV-Vis spectroscopy was used to investigate the binding interaction between the DSP crosslinker and the chloride ionophore. Supplementary Figure S1 shows the peak absorbance of the DSP crosslinker at peak at 285 nm (shown in blue). The covalent binding of the ionophore to the crosslinker causes a shift in the wavelength to 324 nm, as illustrated by the red line in Supplementary Figure S1.

### 3.2. Effect of Buffer pH on Surface Charge of the Capture Probe

The selected chloride ionophore has been reported to be electrically neutral in a work by Xiao et al. (Xiao et al., 1997). This was validated in the zeta potential measurements. It was found that the zeta potential value associated with the surface charge of the ionophore over the entire physiologically relevant range of sweat pH (2 to 8) clustered around 0 mV (Figure 3) and falls within the neutral or near neutral range of −10 mV to 10 mV for particles of nanoscale dimensions [39]. At low pH, the zeta potential value is nearly −2.26 mV, which becomes more negative and stabilizes at ~–10 mV at higher pH. This further emphasizes that the electrical neutrality of the ionophore is maintained despite pH variation, and that the binding chemistry between the analyte and the capture probe is driven by H-bonds and not by electrostatic interaction between the two.

### 3.3. Sensor Calibration and Evaluation of Sensor Performance by EIS and CA

In order to ensure that the proposed sensor caters to chloride sensing of a broad spectrum of pathophysiology, ranging from something as common as dehydration to life-threatening diseases such as cystic fibrosis. Thus, a universal dynamic range of 10–100 mM chloride was aimed for. The calibration dose–response (CDR) values for EIS measurements were reported as the relative percentage change in the modulus of the impedance value at 100 Hz from the baseline/zero dose value. Owing to the observed high SNR and the feasibility of incorporation into a POC or wearable, 100 Hz was our obvious choice. The percentage change in the peak capacitive current value with respect to the zero dose was extracted as output for the CDR from the recorded chronoamperograms. A percentage change of 10 to 40 was observed in the EIS-calibrated dose response (Figure 5a) in relation to dosing from the 10 mM to 100 mM range, while a corresponding relative percentage change of 30 to 80 was obtained from the CA data (Figure 5b). From the slope and R^2^ values of both the CDRs, it can be concluded that the sensitivity technique for detecting biomolecular interaction at the EDL of the EIS system (R^2^ = 0.9746, n = 5) is greater than the CA method (R^2^ = 0.9403, n = 4). The sensor response for the lowest chloride dose was easily discernible from the buffer noise and was well above the specific signal threshold (Supplementary Figure S11). The specific signal threshold for the EIS analysis was defined as three times the ratio of the standard deviation of modulus of impedance to the mean for the replicates of the blank buffer at 100 Hz [40].

### 3.4. Dynamic Chloride Sensing and Discussion of Continuous Data

**Figure 6:**
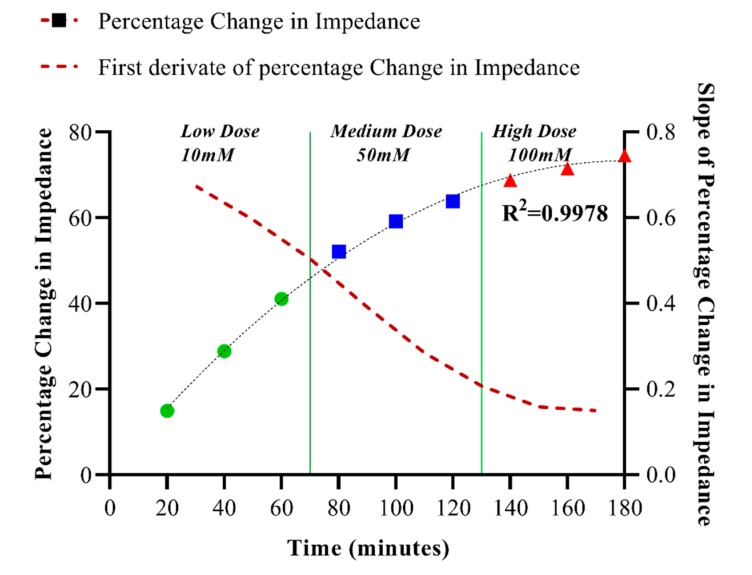
The continuous chloride monitoring capability of the sensor was evaluated by successively dosing with low, medium, and high chloride solutions (3 µL) for a duration of 180 min. Dose-dependent response (R^2^ = 0.9978) was obtained, and the first derivative of the response indicates that the sensor does not saturate even after 3 h.

The continuous monitoring efficacy of the proposed sensing scheme was evaluated by continuously dosing low (10 mM), medium (50 mM), and high (100 mM) levels of chloride for a 180-min duration (n = 3), as shown in Figure 6. A dose-dependent variation in the slope was observed over the ranges of low, medium and high doses (Figure 6). Upon polynomial curve fitting, an R^2^ value of 0.9978 was achieved. In this study, the sensor has been dosed with increasing chloride levels after every 20 min for a span of 3 h. The sensor operated in an accumulative mode, and the sensor’s response to repeated increasing doses has been tracked. The slope of the response has been chosen to discriminate between the low, medium, and high chloride levels. As can be seen from Figure 6, the slope of the curve linearly decreases with increasing chloride doses. The plot reveals that even after 180 min, the sensor response does not completely flatten out, meaning that sensor does not saturate even after 3 h, and that there are still a lot of unbound capture probe sites available, owing to big size difference between the ionophore and analyte.

### 3.5. Testing Sensor Performance on Human Subjects

**Figure 7:**
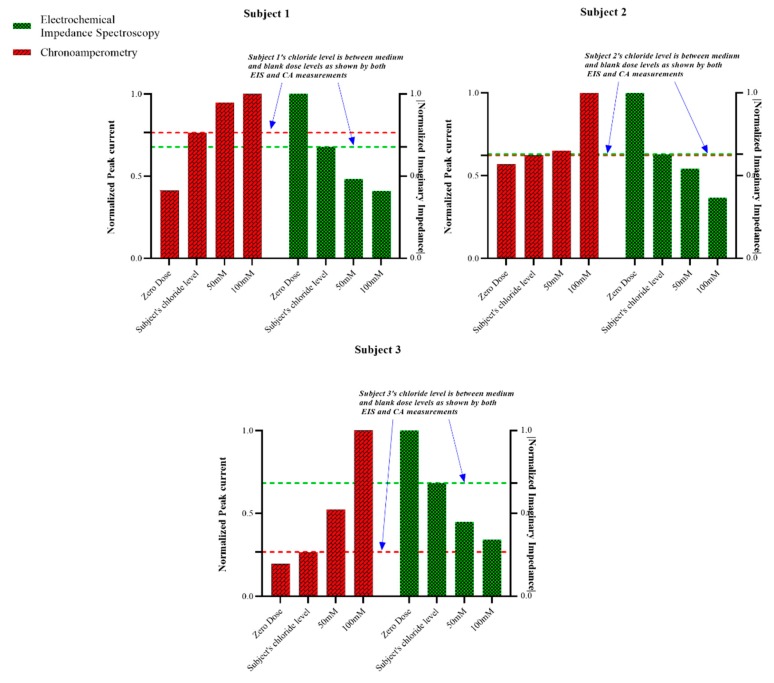
On-body tests for three human subjects (n = 3) done by EIS (100 Hz) and Chronomaperometry indicate that subjects 1, 2 and 3 were healthy.

To test for the measurement efficacy for on-body testing, non-faradaic EIS (100 Hz) and non-faradaic CA measurements were run on three healthy human subjects for three technical replicates each. A blank dose of human sweat analogue, devoid of chloride ions, was taken as negative control, and the highest dose of the sensor dynamic range was chosen as the system validating positive control. As shown in Figure 7, the data obtained from all three subjects confirm that the subjects did not have abnormal chloride levels, as the chloride levels for all of the three subjects fall between the zero (0 mM) and medium dose (50 mM).

## 4. Discussion

In this work, we have demonstrated for the first time an impedance-based affinity biosensor for the detection of chloride ion levels in ultra-low volumes (1–3 microliters) of human sweat, without any active stimulation of sweat glands. By leveraging the surface area to volume enhancement afforded by working at the nanoscale and optimizing the electrode area based on natural sweat elution rates, chloride sensing in the entire physiologically relevant range (10–100 mM) was achieved. To achieve a high sensitivity of chloride quantification in low analyte detection volumes, non-faradaic electrochemical impedance spectroscopy (EIS), a powerful AC-based electroanalysis technique, was used to transduce the modulation of the interfacial properties (charge transfer resistance and double-layer capacitance) of the electrical double layer at the electrode–sweat buffer interface. As a method control, non-faradaic chronoamperometry (CA), a DC-based electroanalytical technique, was used to study the sensor response, and the results from both the methods were compared. The sensor shows good sensitivity and linear calibration dose response (R^2^ >0.94) for both the EIS and CA over the wide linear dynamic range of 10–100 mM. It was found that EIS afforded finer calibration, better resolution, and wider output dynamic range than chronoamperometry. The surface charge behavior of the ionophore as a capture probe was studied by zeta potential measurements for the entire physiological sweat pH range (2 to 8). It was found that the capture probe remains electrically neutral throughout the entire pH range, meaning that the capture probe performance is not impacted by electrostatic crosstalk. The binding chemistry between the capture probe and the analyte was validated by the optical characterization scheme of UV-Vis spectroscopy. Bode and Nyquist plots were obtained for varying chloride doses to inspect the sensor behavior over a wide frequency spectrum of 1 Hz to 1 MHz. The behavior of the electrical double layer at the electrode–sweat buffer interface was modeled as an equivalent electrical circuit, and linear dose-dependent responses for the charge transfer resistance and double-layer capacitance were observed. Since the sensing scheme is envisioned to be incorporated in a continuous monitoring wearable hydration monitoring device, the dynamic response of the sensor to continuous dosing over a span of three hours was studied, and it was found that the sensor output was capable of following the input without saturation for the whole timespan. Finally, based on on-body testing on human subjects, it was validated that the sensor is successfully capable of sensing chloride ion levels in ultra-low sweat volumes eluted at natural sweat rates passively without any active stimulation of sweat glands. The sensor does not rely on iontophoretic, chemical, or exercise-induced stimulation schemes, and is thus very suitable for hydration level monitoring in infants, elderly, immunocompromised patients on chronic medication, and other physically inactive population. The use of ultra-low sweat volumes of sweat make it an ideal sensor for both hypo and hyperchloremia that can be tailored to universally cater to a gamut of disorders ranging from CF to dehydration, all of which can be monitored conveniently in the comfort of one’s home.

## Supplementary Materials

The following are available online at https://www.mdpi.com/1424-8220/19/20/4590/s1. Figure S1: UV-Vis spectra for Crosslinker molecule DSP (blue line) and the ionophore-DSP complex (red line) upon binding. Figure S2: Open Circuit Potential measurement of the gold microelectrode system in aqueous buffer. Figure S3: Bode magnitude plot (modulus of impedance vs Frequency) obtained for crosslinker (DSP), chloride ionophore (ClIP), Zero dose (ZD or 0 mM), 10 mM (Low Dose), 50 mM (Medium Dose) and 100 mM (High Dose) of KCl in Synthetic sweat buffer (pH 6). Figure S4: Bode phase plot (phase of impedance vs Frequency) obtained for crosslinker (DSP), chloride ionophore (ClIP), Zero dose (ZD or 0 mM), 10 mM (Low Dose), 50 mM Medium Dose) and 100 mM (High Dose) of KCl in Synthetic sweat buffer (pH 6). Figure S5: Nyquist plot (imaginary vs real component of impedance) obtained for Zero dose (ZD or 0 mM), 10 mM (Low Dose), 50 mM Medium Dose) and 100 mM (High Dose) of KCl in Synthetic sweat buffer (pH 6). Figure S6: Switch in equivalent electrical circuit upon chloride dosing (KCl dosing in Deionized (DI) water). Figure S7: Bode magnitude plot (modulus of impedance vs Frequency) obtained for Zero dose (ZD or 0 mM), 10 mM (Low Dose), 50 mM Medium Dose) and 100 mM (High Dose) of KCl in DI water. A switch of interfacial properties upon chloride dosing is evident. Figure S8: Bode phase plot (phase of impedance vs Frequency) obtained for Zero dose (ZD or 0 mM), 10 mM (Low Dose), 50 mM Medium Dose) and 100 mM (High Dose) of KCl in DI water. A switch of interfacial properties upon chloride dosing is evident. Figure S9: Nyquist plot (imaginary vs real component of impedance) obtained for Zero dose (ZD or 0 mM), 10 mM (Low Dose), 50mM Medium Dose) and 100 mM (High Dose) of KCl in DI water. A switch of interfacial properties upon chloride dosing is evident. Figure S10: Chronoamperometry plot obtained for Zero dose (ZD or 0 mM), 10 mM (Low Dose), 50 mM Medium Dose) and 100 mM (High Dose) of chloride in human sweat (pH 5.89). Figure S11: Signal and Noise levels for sensor response to chloride doses spiked in synthetic sweat. The response for the lowest chloride dose is clearly discernible from the noise level and is well above the Specific Signal Threshold Level. Figure S12: Slope vs Concentration for the proposed chloride sensor. The observed sensitivity is high in the healthy (low) physiological chloride range.
