# Supplementary material for: Passively Addressable Ultra-Low Volume Sweat Chloride Sensor
Source: Sensors (Basel). 2019 Oct 22;19(20):4590. doi: 10.3390/s19204590 (PMC6833906; doi:10.3390/s19204590)
Supplement: Supplementary file 1 [file sensors-19-04590-s001.pdf]

Passively Addressable Ultra-Low Volume Sweat Chloride Sensor

Antra Ganguly[a], and Shalini Prasad[a*]

[a]Department of Bioengineering, University of Texas at Dallas, Richardson, TX 75080

[*]Corresponding author, Email: shalini.prasad@utdallas.edu

Supplementary information

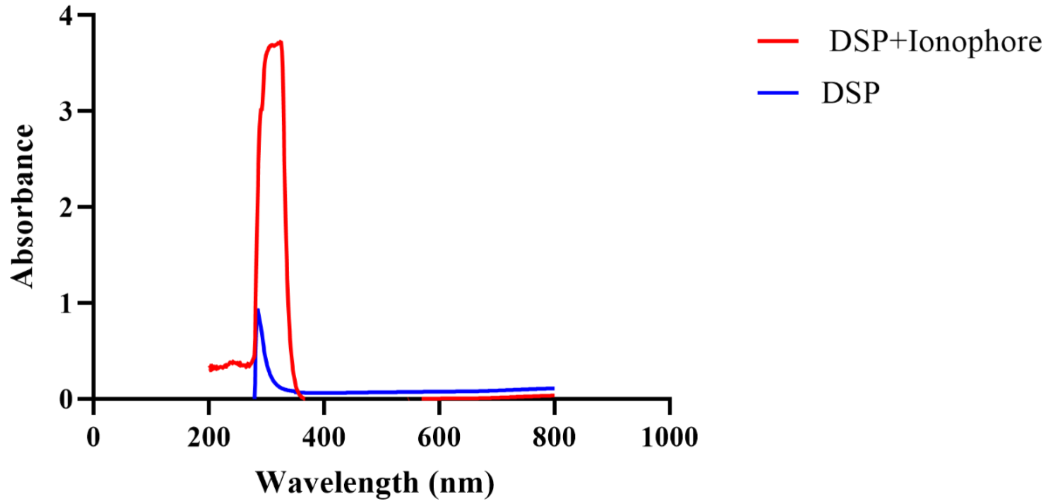

Figure S1: UV-Vis spectra for Crosslinker molecule DSP (blue line) and the ionophore-DSP complex (red line) upon binding

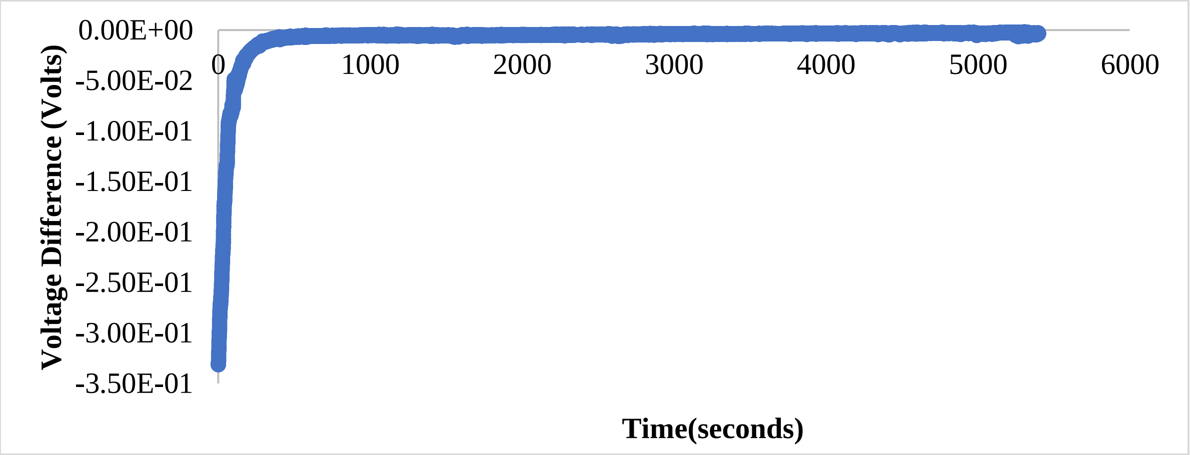

Figure S2: Open Circuit Potential measurement of the gold microelectrode system in aqueous buffer

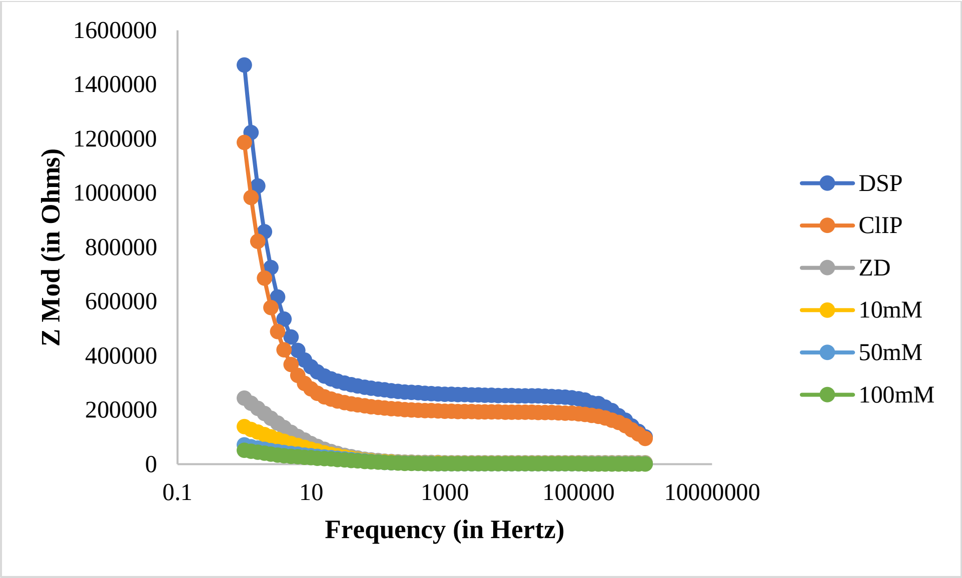

Figure S3: Bode magnitude plot (modulus of impedance vs Frequency) obtained for crosslinker (DSP), chloride ionophore (ClIP), Zero dose (ZD or 0mM), 10mM (Low Dose), 50mM (Medium Dose) and 100 mM (High Dose) of KCl in Synthetic sweat buffer (pH 6).

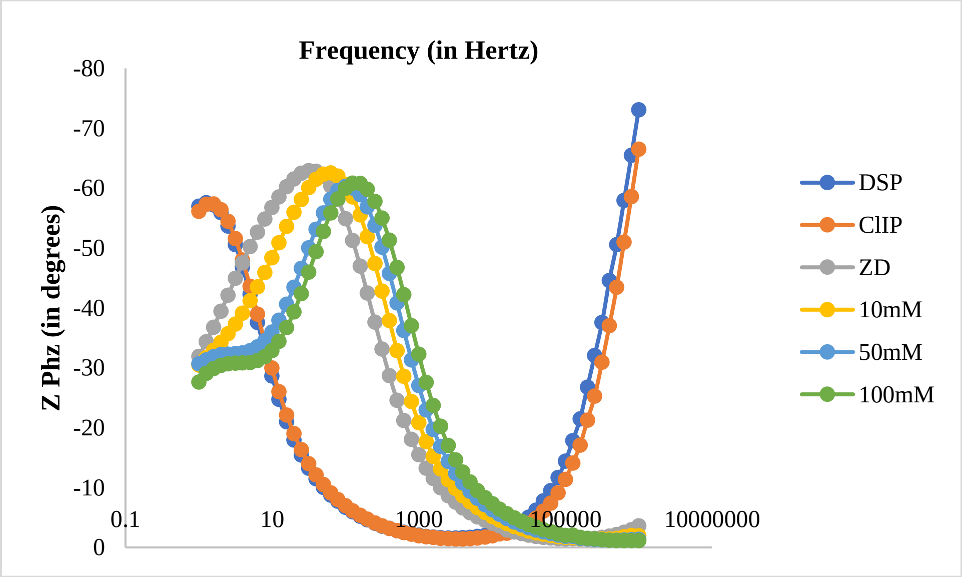

Figure S4: Bode phase plot (phase of impedance vs Frequency) obtained for crosslinker (DSP), chloride ionophore (ClIP), Zero dose (ZD or 0mM), 10mM (Low Dose), 50mM Medium Dose) and 100 mM (High Dose) of KCl in Synthetic sweat buffer (pH 6).

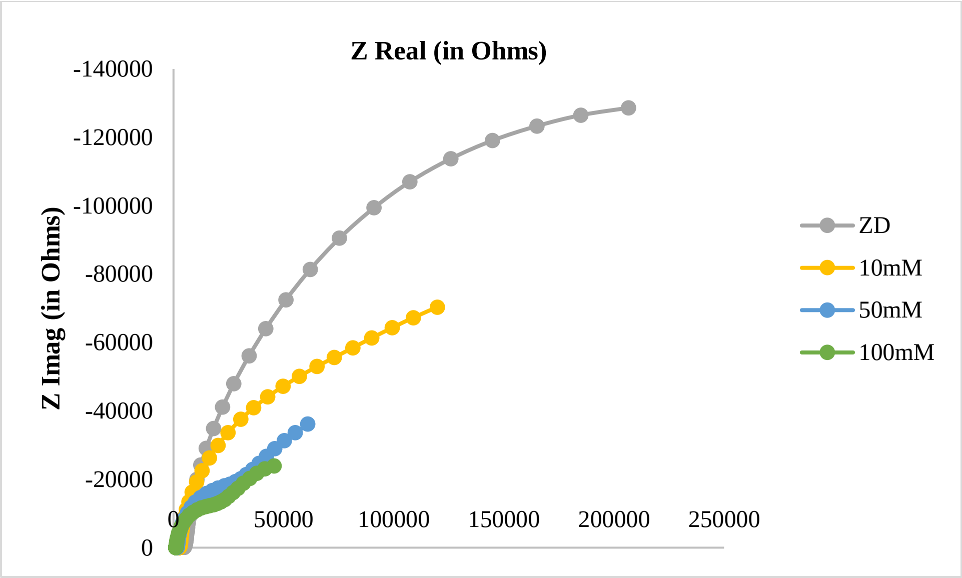

Figure S5: Nyquist plot (imaginary vs real component of impedance) obtained for Zero dose (ZD or 0mM), 10mM (Low Dose), 50mM Medium Dose) and 100 mM (High Dose) of KCl in Synthetic sweat buffer (pH 6).

Circuit for Zero Dose- Chloride Ionophore on DSP (DMSO buffer)

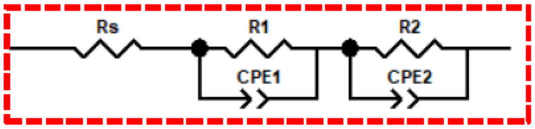

Circuit for KCl Doses- 10, 50 and 100 mM (DI Water buffer)

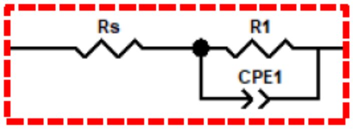

Figure S6: Switch in equivalent electrical circuit upon chloride dosing (KCl dosing in Deionized (DI) water)

***R_s**: Resistance of the solution/buffer.*

***CPE1 and CPE2**: Constant Phase elements 1 and 2(imperfect or leaky capacitor)*

***R1 and R2**: Charge transfer resistances.*

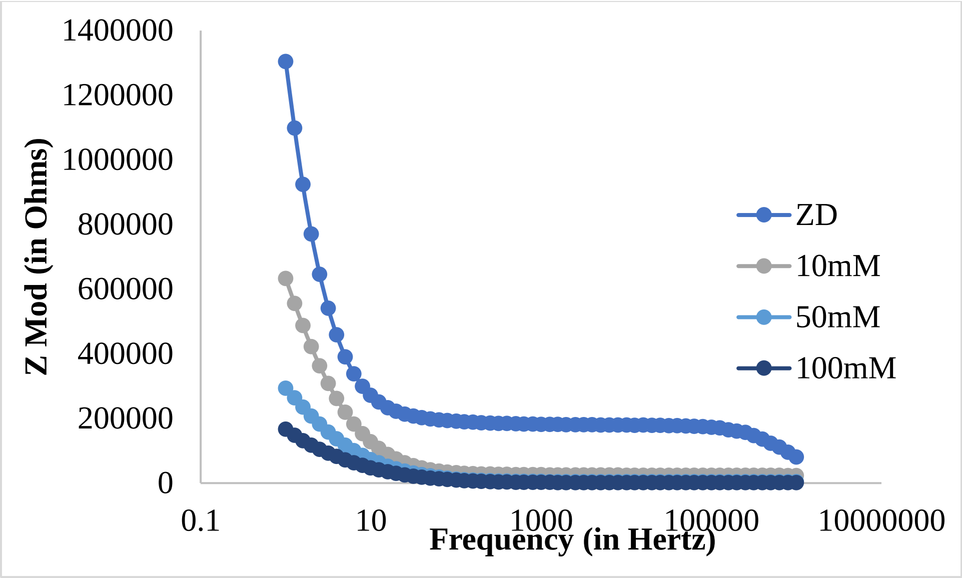

Figure S7: Bode magnitude plot (modulus of impedance vs Frequency) obtained for Zero dose (ZD or 0mM), 10mM (Low Dose), 50mM Medium Dose) and 100 mM (High Dose) of KCl in DI water. A switch of interfacial properties upon chloride dosing is evident.

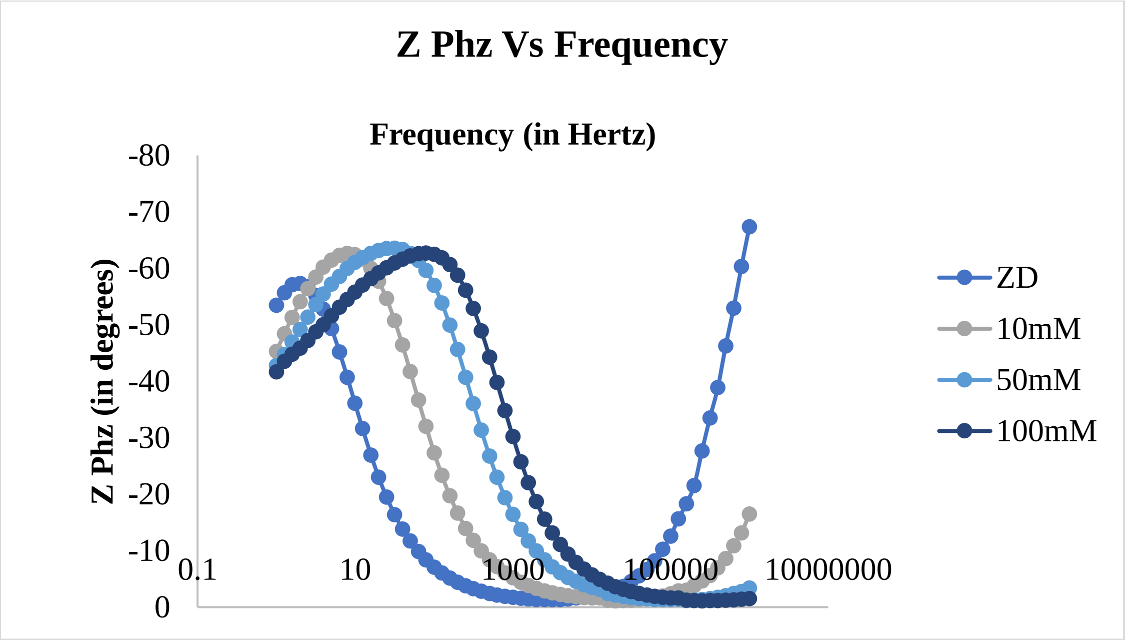

Figure S8: Bode phase plot (phase of impedance vs Frequency) obtained for Zero dose (ZD or 0mM), 10mM (Low Dose), 50mM Medium Dose) and 100 mM (High Dose) of KCl in DI water. A switch of interfacial properties upon chloride dosing is evident.

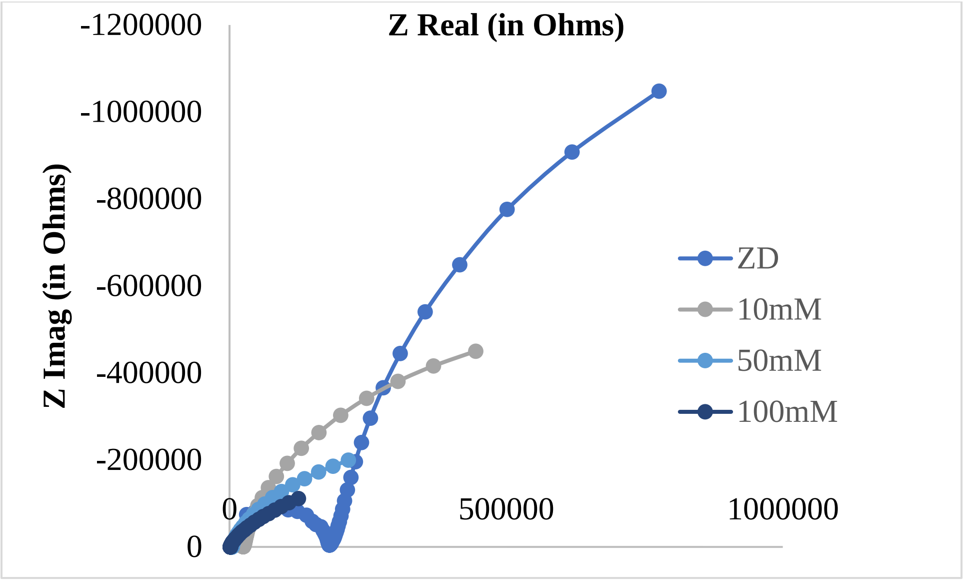

Figure S9: Nyquist plot (imaginary vs real component of impedance) obtained for Zero dose (ZD or 0mM), 10mM (Low Dose), 50mM Medium Dose) and 100 mM (High Dose) of KCl in DI water. A switch of interfacial properties upon chloride dosing is evident.

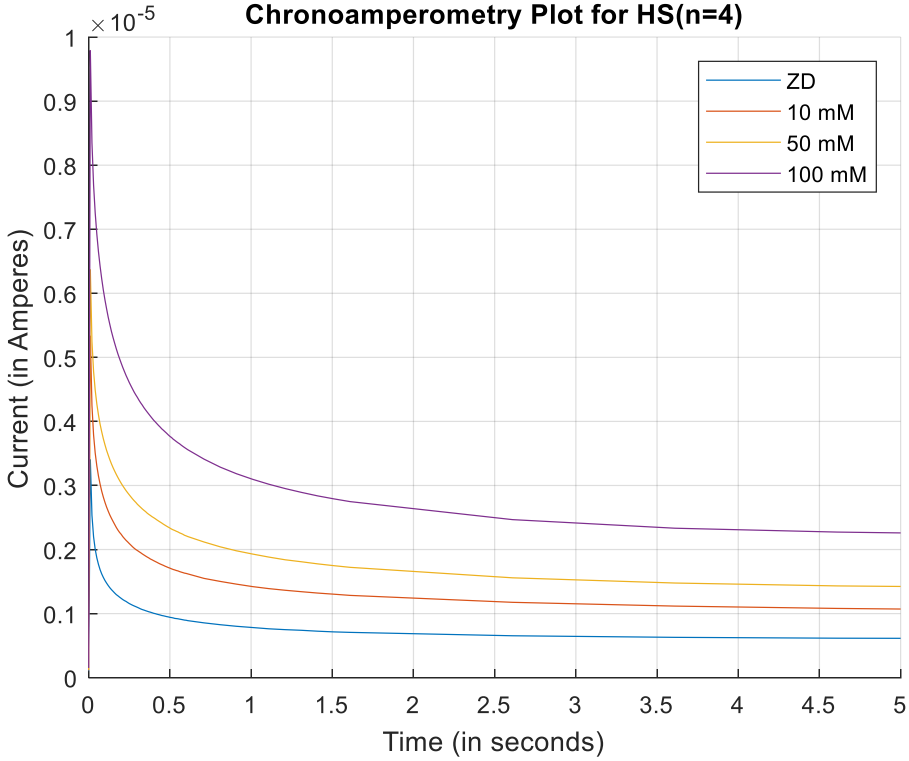

Figure S10: Chronoamperometry plot obtained for Zero dose (ZD or 0mM), 10mM (Low Dose), 50mM Medium Dose) and 100 mM (High Dose) of chloride in human sweat (pH 5.89).

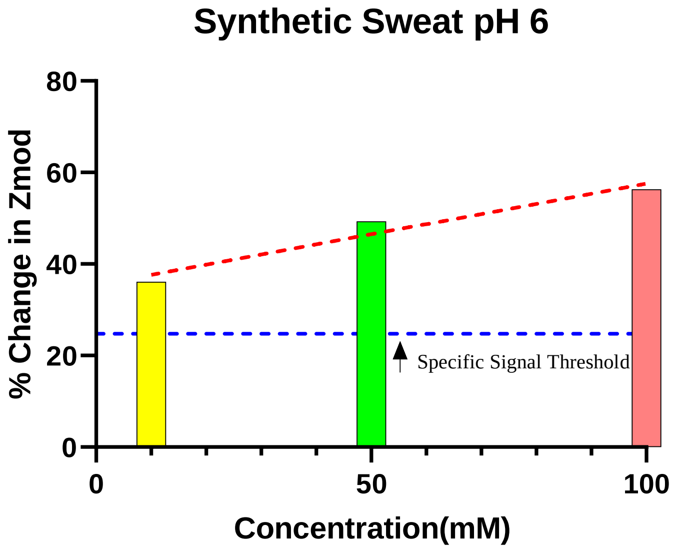

Figure S11: Signal and Noise levels for sensor response to chloride doses spiked in synthetic sweat. The response for the lowest chloride dose is clearly discernible from the noise level and is well above the Specific Signal Threshold Level.

Specific Signal Threshold (SST) was estimated by measuring the replicates of the blank buffer sample using the following formula:

$$\mathbf{SST=(3(SD_{blank})/Mean_{blank})*100}$$

where, $Mean_{blank}$ is the average Zmod of the blank buffer solution and SD_{blank} is the standard deviation of the blank buffer solution.

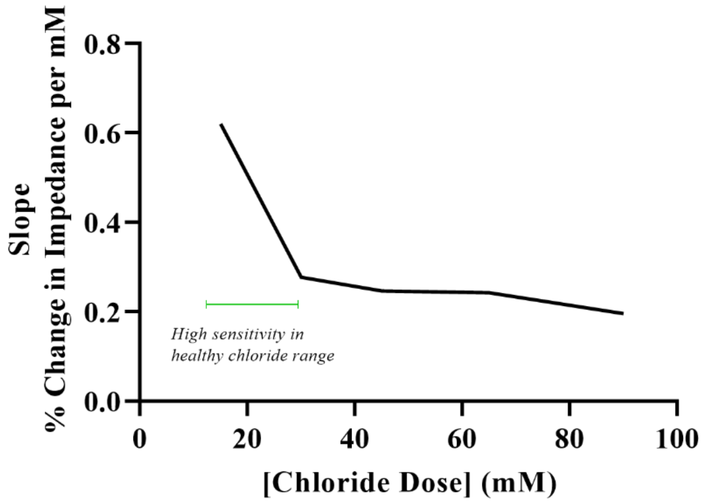

Figure S12: Slope vs Concentration for the proposed chloride sensor. The observed sensitivity is high in the healthy (low) physiological chloride range.

Ion selective electrodes are traditionally used as potentiometric sensors and suffer from equilibration issues between the test and reference solution over time resulting in measurement error. Potentiometric ISEs follow the Nernst equation (logarithmic dependence between potential and activity) and hence suffer from low sensitivity. While such sensors perform well for wide concentration ranges, poor sensitivity is observed for narrow physiologically relevant ranges. The average sensitivity of the proposed sensor response is 0.2648 i.e. 0.2648 % change in Zmod per mM change in chloride concentration. The healthy (i.e. low) chloride range falls between 10-35 mM. The slope/ sensitivity is maximum in this range. Hence, the sensor has significant resolution in that range, which is not the case for ion-selective electrodes as they are tuned for threshold detection and are largely incapable of resolving small concentration changes within this low range.

References

1. Bard, A J, Faulkner, L.R., Leddy, J., Zoski, C.G., 1980. Electrochemical methods: fundamentals and applications. wiley New York.
2. Scholz, F, 2010. Electroanalytical methods. Springer.
3. Randviir, E.P., Banks, C.E., 2013. Analytical Methods. 5, 1098-1115.
4. Daniels, J.S., Pourmand, N., 2007. Electroanalysis: An International Journal Devoted to Fundamental and Practical Aspects of Electroanalysis. 19, 1239-1257.
5. Orazem, M.E., Tribollet, B., 2009. Angew.Chem.Int.Ed. 48, 1532-1534.
6. Armbruster, D.A. and Pry, T., 2008. Limit of blank, limit of detection and limit of quantitation. The clinical biochemist reviews, 29(Suppl 1), p.S49.
7. Choi, D.H., Kim, J.S., Cutting, G.R. and Searson, P.C., 2016. Wearable potentiometric chloride sweat sensor: the critical role of the salt bridge. Analytical chemistry, 88(24), pp.12241-12247.
8. Han, T., Mattinen, U. and Bobacka, J., 2019. Improving the sensitivity of solid-contact ion-selective electrodes by using coulometric signal transduction. ACS sensors, 4(4), pp.900-906.
